# Prospective validation of an AI software for detecting clinically significant prostate cancer on biparametric MRI

**DOI:** 10.1186/s13244-025-02199-9

**Published:** 2026-01-26

**Authors:** Mohammed R. S. Sunoqrot, Rebecca Segre, Gabriel A. Nketiah, Petter Davik, Torill A. E. Sjøbakk, Sverre Langørgen, Mattijs Elschot, Tone F. Bathen

**Affiliations:** 1 Department of Circulation and Medical Imaging, Norwegian University of Science and Technology (NTNU), Trondheim, Norway; 2 Department of Radiology and Nuclear Medicine, St. Olavs Hospital, Trondheim University Hospital, Trondheim, Norway; 3 Department of Urology, St. Olav’s Hospital, Trondheim University Hospital, Trondheim, Norway; 4 Department of Clinical and Molecular Medicine, Norwegian University of Science and Technology (NTNU), Trondheim, Norway

**Keywords:** Prostate cancer, Magnetic resonance imaging, Artificial intelligence, Software, Prospective study

## Abstract

**Objectives:**

To evaluate the feasibility and safety (primary endpoints), and performance (secondary endpoint) of a new artificial intelligence (AI) software for detecting clinically significant prostate cancer (csPCa) on biparametric MRI (bpMRI) compared to an expert radiologist.

**Materials and methods:**

In this prospective study at St. Olavs Hospital, Norway (December 2023–October 2024), 89 consecutive biopsy-naïve men underwent bpMRI for suspected PCa. Scans were interpreted by a radiologist using PI-RADS v2.1 and a radiomics-based AI software. Biopsies were obtained from all radiologist- and/or AI-identified lesions. csPCa was defined as ISUP ≥ 2. Feasibility was defined by a < 10% software-failure rate, and safety by the absence of serious adverse device effects (SADEs). Performance was evaluated with ROC, free-response ROC, and precision-recall curves.

**Results:**

Among 89 patients eligible for primary endpoints evaluation, the software demonstrated feasibility (7% failure rate) and safety (no SADEs). Among 76 eligible for secondary endpoint evaluation (median age 68 years [IQR: 63–73]), csPCa was found in 51% (39/76). Patient-level, software achieved an area under the ROC curve [95% CI] of 0.90 [0.83, 0.96] versus 0.86 [0.76, 0.93] (*p* = 0.25). At a retrospectively optimized threshold matching the radiologist’s patient-level sensitivity at PI-RADS 3 (0.92), software achieved specificity of 0.68 [0.57, 0.78] versus 0.57 [0.46, 0.68] (*p* = 0.29). Lesion-level, software achieved higher average precision (0.61 [0.52, 0.71] vs. 0.56 [0.46, 0.67]) and lower average false-positive per patient (0.33 [0.22, 0.43] vs. 0.41 [0.30, 0.52]) at the optimized threshold.

**Conclusion:**

The software was feasible and safe, and diagnostic performance showed potential to reduce unnecessary biopsies.

**Critical relevance statement:**

This clinically validated artificial intelligence software enables feasible and safe detection of clinically significant prostate cancer on biparametric MRI, with demonstrated potential to reduce unnecessary biopsies and improve diagnostic accuracy, indicating potential for integration into clinical prostate cancer care.

**Key Points:**

- A fully automated radiomics software for clinically significant prostate cancer detection on biparametric MRI was prospectively clinically validated.
- The software demonstrated feasibility and safety, with potential to reduce unnecessary biopsies and improve diagnostic accuracy.
- The investigated radiomics software has the potential for integration into clinical prostate cancer care.

**Graphical Abstract:**

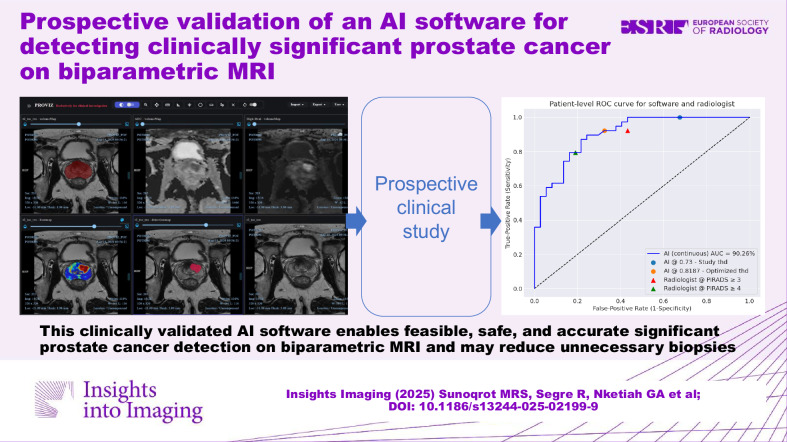

## Introduction

Pre-biopsy multiparametric MRI enhances the detection of clinically significant prostate cancer (csPCa) [1, 2]. However, manual MRI interpretation is time-consuming, variable, and susceptible to false-positives [3–6], which may lead to unnecessary biopsies and overdetection [7].

While the Prostate Imaging-Reporting and Data System (PI-RADS) standardizes prostate MRI interpretation [8] and has improved consistency [9, 10], challenges such as inter-observer variability and false-positives remain concerns [10–14].

As MRI is recommended before prostate biopsy, there is a growing need for efficient and reliable diagnostic solutions [10]. Biparametric MRI (bpMRI) offers a cost-effective alternative to multiparametric MRI with comparable csPCa detection [15], while artificial intelligence (AI) tools may enhance bpMRI interpretation and reduce the limitations of the manual, traditional interpretation [16–18].

Our in-house AI software, described in [19], automates bpMRI analysis and may improve csPCa detection while reducing false-positives and unnecessary biopsies. A recent retrospective study showed performance comparable to expert radiologists [19]. However, prospective clinical validation of AI tools is essential before integration into clinical workflows [17]. Such validation mitigates selection and spectrum bias, enables real-world feasibility and safety assessment, and aligns with regulatory expectations for clinical AI under frameworks such as the EU’s Medical Device Regulation (MDR) and AI Act and the U.S. FDA’s Software as a Medical Device guidance [20–24].

Therefore, we aimed to evaluate the feasibility, safety, and diagnostic performance of our software in a prospective clinical setting. We hypothesize that use of the software is feasible and safe, with an optimized diagnostic performance for detecting csPCa comparable to an expert radiologist.

## Materials and methods

### Study design and patients

This prospective clinical, pilot, single-site study aimed to evaluate the diagnostic feasibility and safety (primary endpoints), and performance (secondary endpoint) of the investigational AI software for detecting csPCa, defined as International Society of Urological Pathology (ISUP) grade group (GG) 2 or higher [25], using bpMRI. The study was conducted consecutively from December 2023 to October 2024 (paused 10–29 January 2024 for protocol refinement) at St. Olav’s hospital, Trondheim University Hospital, Trondheim, Norway, was approved by the Norwegian Ethics Committees for Clinical Trials on Medicinal Products and Medical Devices (REK KULMU; identifier no. 479272) and registered at ClinicalTrials.gov (identifier no. NCT06000046), where the full protocol is accessible. Written informed consent was obtained from all patients. The study adhered to the Declaration of Helsinki ethical principles and complied with STARD guidelines [26]. The funding organizations had no role in this study.

The study comprised a 3-week piloting phase involving 13 patients to ensure software integration and refine the final protocol (see Supplementary Material), followed by the main study phase, which adhered to the finalized protocol and is described here.

**Fig. 1:**
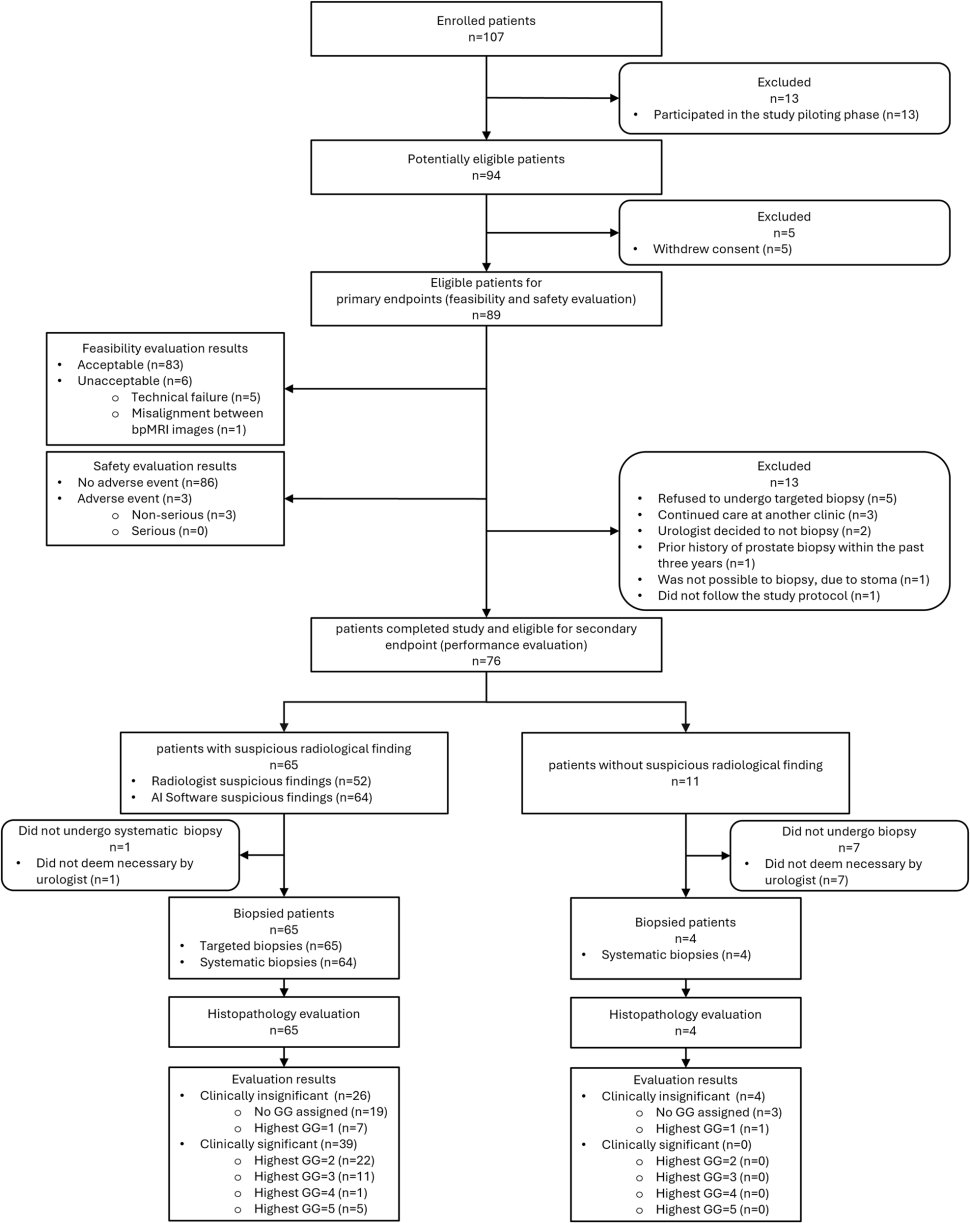
Diagram illustrating the flow of patients throughout the study (software’s study threshold = 0.73). AI, artificial intelligence; GG, International Society of Urological Pathology (ISUP) grade group

Biopsy-naïve adult men (age ≥ 18 years) were eligible for enrollment in the study if they underwent MRI for suspected PCa as part of the Norwegian standardized PCa care pathway at the study site, during the recruitment period from December 2023 to August 2024, due to an elevated prostate-specific antigen (PSA) level (> 3 ng/mL) and/or abnormal digital rectal examination findings, or suspicious incidental findings on other imaging. Exclusion criteria comprised a history of prostate biopsy within the previous 3 years, enrollment in an active surveillance program for PCa, hip replacements that could compromise image quality, claustrophobia, intolerance to glucagon or hyoscine butylbromide, or inability to personally provide informed consent. Patients could withdraw from the study at any time. Patients who left the study site, did not adhere to the study protocol, had contraindications for biopsy, or chose not to complete the study were included in the primary endpoints analysis, but excluded from the secondary endpoint analysis. Patients’ race or ethnicity information was not collected. The patient flow diagram for the study is presented in Fig. 1.

### Image acquisition

All patients underwent bpMRI scanning using a Magnetom Skyra 3-T MRI scanner (Siemens Healthineers) following PI-RADS v2.1 guidelines. Scans were performed with the use of a pelvic phased-array surface coil without an endorectal coil. Detailed acquisition parameters are provided in Table S1.

### AI software

The AI software we used is a fully automated, in-house developed, radiomics-based system, described in [19] and summarized in the Supplementary Material. The model trained on a multicenter dataset of 415 patients (none from the current study site) using an XGBoost classifier. The software generates a csPCa tumor probability map, post-processed into a lesion detection map. To manage false-positives, it retains up to three lesions per patient with the highest csPCa probability scores above a predefined threshold. This design choice was informed by internal risk analysis on retrospective data. Threshold configuration is further described under “Statistical analysis.”

**Fig. 2:**
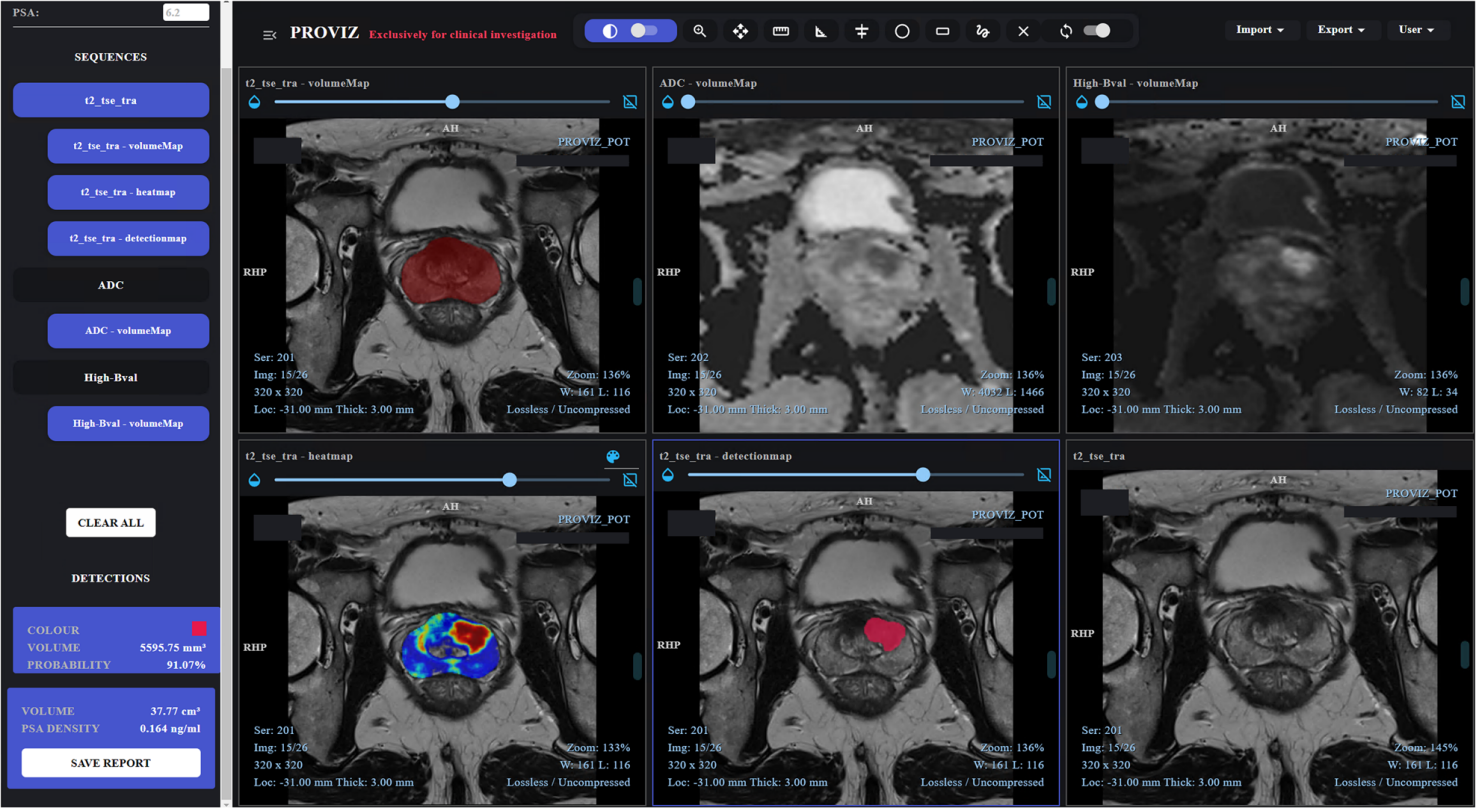
Graphical user interface of the investigated artificial intelligence (AI) software, illustrating the output for a case study. The images represent a 69-year-old patient with a prostate-specific antigen (PSA) level of 6.2 ng/mL. The software detected a potential clinically significant prostate cancer (csPCa) lesion in the anterior transition zone on the mid-left region of the prostate gland, with a probability score of 91.07%. This lesion was independently identified by a radiologist and assigned a Prostate Imaging-Reporting and Data System (PI-RADS) version 2.1 score of 5. The software output includes the following planes (top-left to bottom-right): (1) T2-weighted (T2W) image overlaid with an automatically generated prostate gland segmentation mask, (2) co-registered apparent diffusion coefficient (ADC) map, (3) co-registered diffusion-weighted high b-value (HBV) image, (4) T2W image overlaid with the software-generated csPCa probability map, (5) T2W image overlaid with the software-generated lesion detection map, and (6) T2W image without overlays

The software was accessed via a desktop graphical user interface integrated with a secure, study-specific picture archiving and communication system (PACS). Pseudonymized bpMRI scans were transferred from hospital PACS to the study server via teleradiology, processed automatically, and returned results for review through the interface. Figure 2 shows an example output; server setup details are in Table S2.

### Study procedures

**Fig. 3:**
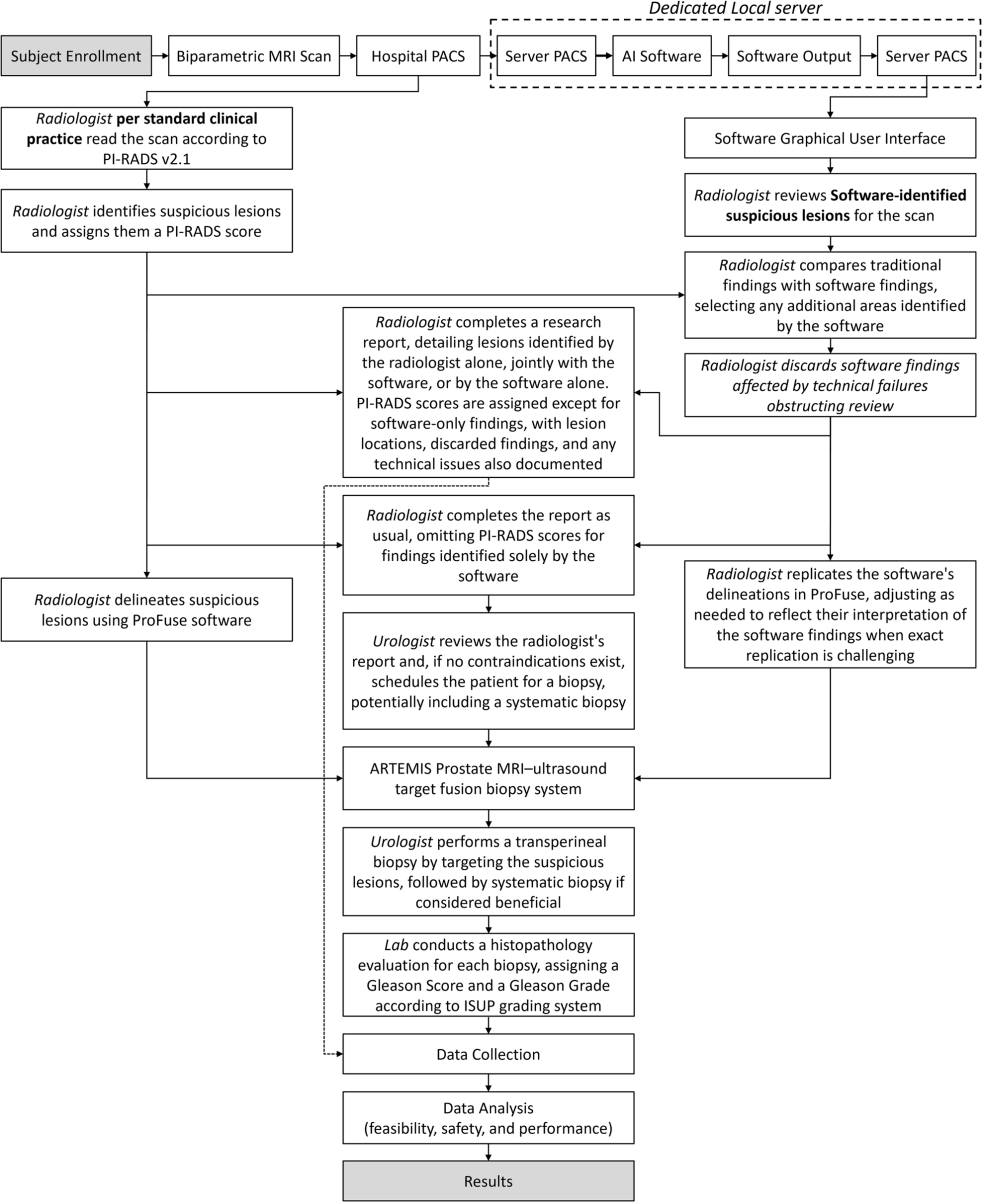
Overview of the study workflow. AI, artificial intelligence; ISUP, International Society of Urological Pathology; PACS, picture archiving and communication system; PI-CAI, Prostate Imaging-Reporting and Data System

The study workflow is illustrated in Fig. 3. All patients underwent bpMRI scans, with images stored in the hospital PACS. Clinical data, including patient age, PSA levels, digital rectal examination findings, and family history, were collected at the time of the urology assessment and recorded in an electronic case report form. To ensure confidentiality, scans were deidentified and pseudonymized and then transferred via teleradiology to the study PACS server for processing by the AI software.

An expert radiologist (S.L.; read > 5000 cases [27]) with over 15 years of experience in prostate MRI interpretation, blinded to the software outputs and with access to clinical data, initially reviewed each bpMRI scan using the PI-RADS v2.1 criteria [8] as per standard clinical practice. PI-RADS ≥ 3 was considered suspicious for csPCa. Subsequently, the radiologist accessed the software findings through a user interface at their workstation, comparing these with their own evaluations. Software findings impacted by technical failures that obstructed radiological review were discarded. Each lesion was categorized into one of three groups: identified by the radiologist alone, by both the radiologist and the software, or by the software alone. In addition, lesion locations and PI-RADS scores, if present, were recorded in the report form.

To prepare for targeted biopsies, the prostate gland and all suspicious lesions were delineated by the radiologist using Eigen Artemis ProFuse software (v2; Eigen Health) and exported to the Eigen Artemis MRI–ultrasound prostate fusion biopsy system (v3.5). For software-identified lesions, the radiologist manually replicated the software-generated delineations in ProFuse, with adjustments as needed, to ensure their transfer for biopsy planning. A urologist (one of 7, with 1–10 years of experience) reviewed the radiological report to confirm biopsy eligibility, ensuring no contraindications. During the biopsy procedure, the delineations were imported into the fusion system without blinding to their source. A transrectal ultrasound scan was performed and fused with MRI data to guide biopsies. All delineated PI-RADS or software lesions underwent transperineal targeted biopsies (1–5 cores). Systematic biopsies (1–10 biopsies [1–3 cores]) were additionally performed for these patients unless the urologist deemed them unnecessary per site protocol. Patients without suspicious findings by PI-RADS and/or software underwent systematic biopsy only when indicated by other risk factors or clinical concerns (e.g., family history), per the same protocol. Biopsy samples were labeled with lesion numbers and PI-RADS locations before being sent for histopathological analysis. Patients were followed up for 3 weeks post-biopsy to monitor for adverse events.

In the histopathology laboratory, pathologists, blinded to whether the targeted lesion was identified by the radiologist or the software, analyzed biopsy samples separately for each lesion, including those obtained from systematic biopsies, in compliance with ISUP guidelines [25]. For lesions with multiple samples, results were pooled to determine an overall GG. ISUP GG1 was classified as clinically insignificant PCa, while GG2-5 were classified as csPCa.

At the study conclusion, all data were collected and analyzed in compliance with the General Data Protection Regulation within a secure, ISO-certified cloud environment on Ubuntu 22.04.5 LTS. Indeterminate or inconsistent data were resolved in consultation with the relevant clinician(s), and patients with incomplete data were excluded from analysis.

### Endpoints

The primary endpoints were the feasibility and safety of the AI software, with performance as a secondary endpoint. Feasibility was defined as technical issues (i.e., poor gland segmentation or co-registration, or software deficiencies obstructed radiological review), occurring in < 10% of scan interpretations. The 10% failure rate was based on internal feasibility expectations deemed appropriate for this pilot study. Safety was monitored by tracking serious adverse device effects (SADEs), defined under MDR Article 2(58) [23], with the software considered safe if none were attributable to it. SADEs could potentially arise if software-prompted biopsies, missed by the radiologist, led to complications such as infection or bleeding, resulting in serious harm. Performance was evaluated by comparing the software detection rate of csPCa lesions to that of an expert radiologist, and was considered acceptable if it was similar or higher than the radiologist’s.

### Statistical analysis

The aim was to enroll 80 patients in this pilot study, thus approximately 20% of the annual bpMRI scans at the study site. This number was adequate for the primary endpoints, allowing estimation of a 10% software-failure rate with an exact 95% confidence interval (95% CI) of approximately 4–17% to support a go/no-go decision. The secondary endpoint (diagnostic performance) was prespecified as exploratory and not formally powered.

The software-failure rate was calculated as the proportion of technical failures reported by radiologists in report forms, while safety was assessed by the number of SADEs recorded.

Compared to the original development study by Nketiah et al [19], a relatively low software probability threshold was applied to invoke targeted biopsies (software’s study threshold = 0.73). This prioritized sensitivity and accepted a higher false-positive rate to minimize the risk of missed csPCa in this pilot and to enable analysis of software performance at a retrospectively optimized detection threshold.

Diagnostic performance was evaluated using receiver operating characteristic (ROC), precision-recall and free-response ROC (FROC) analyses. The area under the ROC curve (AUC) and average precision (AP) were calculated to assess overall performance at the patient-level and lesion-level, respectively. To enable comparison with the radiologist at a clinically relevant operating point, an optimized software threshold was established by matching the patient-level radiologist sensitivity at the PI-RADS ≥ 3 threshold (software’s optimized threshold = 0.8187), prespecified as exploratory and not used for clinical decision-making. This threshold was subsequently applied to lesion-level analyses, which included patients with targeted biopsies or positive systematic biopsies. Further diagnostic performance metrics included accuracy, precision, and error rates. 95% CIs for AUC and AP were estimated using 1000 bootstrap resamples, while CIs for other metrics used the standard normal approximation [28]. AUCs were compared with DeLong’s test; paired proportions were compared with McNemar’s exact test; two-sided α = 0.05. All analyses were conducted using Python (v3.11.0) by M.R.S.S. and R.S. Analysis code can be found at https://github.com/ntnu-mr-cancer/POT-Analysis.

## Results

### Patient characteristics

A total of 107 men were assessed for eligibility for participation in the study. After excluding 18 patients enrolled during the piloting phase (*n* = 13) or who withdrew their consent (*n* = 5), 89 patients remained eligible for primary endpoints evaluation. Among the patients that completed the study and were also eligible for secondary endpoint evaluation (*n* = 76), the median [IQR] age was 68 [63–73] years, with a median PSA level of 8.0 [5.9–11.7] ng/mL and a PSA density of 0.14 [0.11–0.28] ng/mL². Radiological evaluation identified 65 (86%) patients as suspicious (51 (78%) by both the radiologist and software, 1 (2%) by radiologist alone, and 13 (20%) by software alone). All 65 underwent targeted biopsies, with 64 (98%) also receiving systematic biopsies. The median [IQR] time from imaging to biopsy was 14 [8–20] days. csPCa was discovered in 39 (51%, 95% CI: [40%, 63%]) patients. Four patients without radiologist or software findings underwent systematic biopsy; none had csPCa. Detailed characteristics of patients and lesions are presented in Table 1.

**Table 1:** Patients and lesion characteristics (*n* = 76)

Characteristic	Value
Age (years)*	68 (63─73)
PSA level (ng/mL)*	8.0 (5.9─11.7)
Prostate volume (cm^3^)*	45 (35─65)
PSA density (ng/mL^2^)*	0.14 (0.11─0.28)
bpMRI to biopsy interval (days)*	14 (8─20)
Family history of PCa	
Negative	26 (34)
Positive	19 (25)
Unknown	31 (41)
DRE findings	
Negative	28 (37)
Positive	29 (38)
Unknown	19 (25)
Radiology findings	
Negative	11 (14)
Positive	65 (86)
Radiologist + AI software	51 (78)
Radiologist alone	1 (2)
AI software alone	13 (20)
Highest PI-RADS score	
< 3	24 (32)
3	14 (18)
4	15 (20)
5	23 (30)
Highest AI software probability score*	0.85 (0.82─0.90)
Number of lesions on bpMRI	125
Radiologist*	1 (0─1)
AI software*	1 (1─2)
Combined	
0 lesions	11 (15)
1 lesion	26 (34)
2 lesions	22 (29)
3 lesions	13 (17)
4 lesions	4 (5)
Number of biopsied patients	69 (91)
Biopsy method (*n* = 69)	
Targeted + systematic	64 (93)
Targeted only	1 (1)
Systematic only	4 (6)
Highest ISUP grade group (*n* = 69)	
No group assigned	22 (32)
1	8 (12)
2	22 (32)
3	11 (16)
4	1 (1)
5	5 (7)
Clinically significant PCa	
Negative	37 (49)
Positive	39 (51)

Unless stated otherwise, the data represent the number of patients, with percentages provided in parentheses

*AI* artificial intelligence, *bpMRI* biparametric MRI, *DRE* digital rectal examination, *ISUP* International Society of Urological Pathology, *PCa* prostate cancer, *PI-RADS* Prostate Imaging-Reporting and Data System, *PSA* prostate-specific antigen

* Values are expressed as medians, with interquartile ranges in parentheses

### Feasibility and safety

Technical issues were encountered in 6 cases (7%, 95% CI: [2%, 12%]). In one case, the radiologist found bpMRI images alignment unacceptable but could still delineate the 2 software findings. In the remaining 5, software deficiencies obstructed radiological review, leading to the discard of one software finding per case due to artifact (*n* = 1), inability to manually delineate (*n* = 3), or small size (*n* = 1).

Additionally, two cases had discarded software findings, while another software finding in each case was biopsied. One was considered a protocol deviation after the radiologist deemed it non-real, while the other was due to image export/import issues in the biopsy fusion system.

No SADEs were reported. Three non-serious adverse events (3% [0%, 7%]) unrelated to the software were recorded: one hospitalization for post-biopsy infection, one case of infection not requiring hospitalization, and one probable vasovagal syncope resulting in brief loss of consciousness post-biopsy.

### Diagnostic performance

**Fig. 4:**
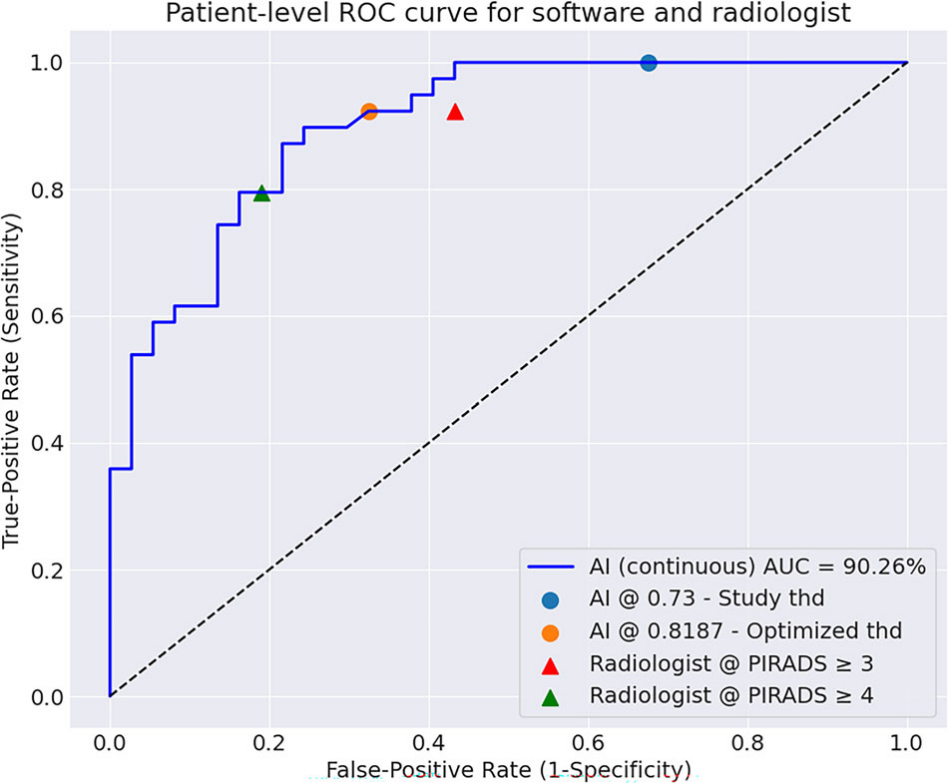
Patient-level receiver operating characteristic (ROC) curve of the artificial intelligence (AI) software, with corresponding Prostate Imaging-Reporting and Data System (PI-RADS) version 2.1 operating points from the radiologist’s assessment. The AI software’s study threshold (0.73) represents the system’s performance during the prospective study prior to optimization. The AI software’s optimized threshold (0.8187) was calibrated to match the radiologist’s sensitivity at the PI-RADS ≥ 3 threshold. The diagonal dashed line denotes the ROC curve of a random classifier, with an area under the curve (AUC) of 0.50

At the patient-level (Fig. 4), the AI software yielded an AUC of 0.90 (95% CI: [0.83, 0.96]), which was modestly, but not significantly higher than the radiologist’s AUC of 0.86 [0.76, 0.93], suggesting comparable diagnostic discrimination between csPCa-positive and csPCa-negative cases (*p* = 0.25). The prespecified retrospective calibration to match the radiologist’s sensitivity of 0.92 [0.86, 0.98] at the PI-RADS ≥ 3 threshold identified an optimized software threshold of 0.8187. At this operating point, the software achieved higher specificity (0.68 [0.57, 0.78] vs. 0.57 [0.46, 0.68]; *p* = 0.29), and improved overall accuracy (0.80 [0.71, 0.89] vs. 0.75 [0.65, 0.85]; *p* = 0.34), although these differences were not statistically significant.

Using the optimized threshold (0.8187), the software would have referred 5% (4/76) fewer patients for biopsy compared to the radiologist. Of the cases uniquely identified by the software (*n* = 3), one (33%) was a true-positive, whereas the radiologist identified 7 unique cases, of which one (14%) was true-positive. Table 2 shows patient-level detection rates stratified by GG, with lower software detection observed for GG 1 and unassigned cases.

**Table 2:** Comparative analysis of patient-level results for radiologist and retrospectively optimized artificial intelligence (AI) software (optimized threshold = 0.8187) detection rates using histopathology as the ground truth

Biopsy result	Radiologist	AI software
Overall		
csPCa	69 [57, 82] (36/52)	75 [63, 87] (36/48)
Non-csPCA	31 [18, 43] (16/52)	25 [13, 37] (12/48)
Highest ISUP grade group		
No group assigned	21 [10, 32] (11/52)	13 [3, 22] (6/48)
1	10 [2, 18] (5/52)	13 [3, 22] (6/48)
2	36 [23, 50] (19/52)	39 [26, 53] (19/48)
3	21 [10, 32] (11/52)	23 [11, 35] (11/48)
4	2 [0, 6] (1/52)	2 [0, 6] (1/48)
5	10 [2, 18] (5/52)	10 [2, 19] (5/48)

Data are presented as percentages with 95% confidence intervals in brackets and the number of patients shown in parentheses

*AI* artificial intelligence, *ISUP* International Society of Urological Pathology, *csPCa* clinically significant prostate cancer

**Fig. 5:**
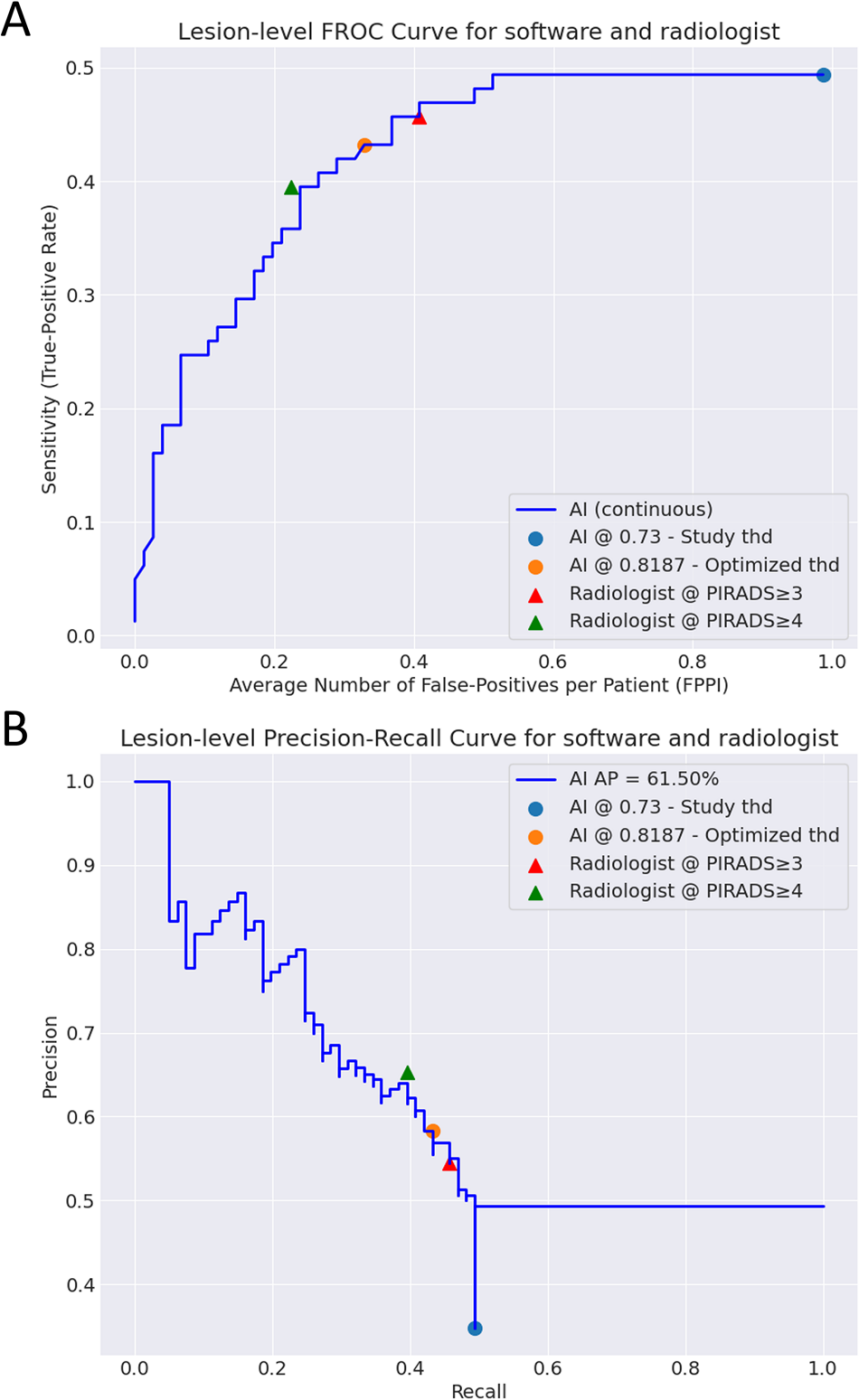
Lesion-level free receiver operating characteristic (FROC) curve (**A**) and precision-recall curve (**B**) of the artificial intelligence (AI) software, with corresponding Prostate Imaging-Reporting and Data System (PI-RADS) version 2.1 operating points from the radiologist’s assessment. The software’s optimized threshold was calibrated to match the radiologist’s sensitivity at the PI-RADS ≥ 3 threshold on the patient-level ROC curve. AP, average precision

At the lesion-level (Fig. 5), the retrospectively optimized software demonstrated a higher AP of 0.62 [0.51, 0.71] compared to the radiologist’s 0.56 [0.46, 0.67]. The FROC analysis showed a lower average false-positive rate per patient for the software (0.33 [0.22, 0.43] vs. 0.41 [0.30, 0.52]), albeit at a slightly reduced sensitivity (0.43 [0.36, 0.51] vs. 0.46 [0.38, 0.53]). Table 3 shows lesion-level detection rates stratified by GG, with both methods performing similarly overall.

**Table 3:** Comparative analysis of lesion-level results for radiologist and retrospectively optimized artificial intelligence (AI) software (optimized threshold = 0.8187) detection rates using histopathology as the ground truth

Biopsy result	Radiologist	AI software
Overall		
csPCa	54 [43, 66] (37/68)	58 [46, 71] (35/60)
Non-csPCA	46 [34, 57] (31/68)	42 [29, 54] (25/60)
Highest ISUP grade group		
No group assigned	29 [19, 40] (20/68)	25 [14, 36] (15/60)
1	16 [7, 25] (11/68)	17 [7, 26] (10/60)
2	29 [19, 40] (20/68)	31 [20, 43] (19/60)
3	15 [6, 23] (10/68)	17 [7, 26] (10/60)
4	3 [0, 7] (2/68)	3 [0, 8] (2/60)
5	7 [1, 14] (5/68)	7 [0, 13] (4/60)

Data are presented as percentages with 95% confidence intervals in brackets and the number of lesions shown in parentheses

*AI* artificial intelligence, *ISUP* International Society of Urological Pathology, *csPCa* clinically significant prostate cancer

A detailed summary of patient- and lesion-level performance metrics is provided in Table 4. Results from all biopsies indicated by the study threshold (0.73) are available in Tables S3 and S4.

**Table 4:** Patient- and lesion-level detection performance metrics for the radiologist at the PI-RADS ≥ 3 threshold and the retrospectively optimized artificial intelligence (AI) software (optimized threshold = 0.8187)

Metric	Radiologist	AI software
Patient-level		
AUC	0.86 [0.76, 0.93]	0.90 [0.83, 0.96]
Sensitivity	0.92 [0.86, 0.98] (36/39)	0.92 [0.86, 0.98] (36/39)
Specificity	0.57 [0.46, 0.68] (21/37)	0.68 [0.57, 0.78] (25/37)
Accuracy	0.75 [0.65, 0.85] (57/76)	0.80 [0.71, 0.89] (61/76)
Precision	0.69 [0.59, 0.80] (36/52)	0.75 [0.65, 0.85] (36/48)
F1 score	0.79 [0.70, 0.88]	0.83 [0.74, 0.91]
True-positives	0.47 [0.36, 0.59] (36/76)	0.47 [0.36, 0.59] (36/76)
False-positives	0.21 [0.12, 0.30] (16/76)	0.16 [0.08, 0.24] (12/76)
True-negatives	0.28 [0.18, 0.38] (21/76)	0.33 [0.22, 0.43] (25/76)
False-negatives	0.04 [0.00, 0.08] (3/76)	0.04 [0.00, 0.08] (3/76)
Lesion-level		
AP	0.56 [0.46, 0.67]	0.61 [0.52, 0.71]
Sensitivity	0.46 [0.38, 0.53] (37/81)	0.43 [0.36, 0.51] (35/81)
Accuracy	0.54 [0.47, 0.62] (89/164)	0.57 [0.49, 0.64] (93/164)
Precision	0.54 [0.47, 0.62] (37/68)	0.58 [0.51, 0.66] (35/60)
F1 score	0.50 [0.42, 0.57]	0.50 [0.42, 0.57]
FPPI	0.41 [0.30, 0.52]	0.33 [0.22, 0.43]
True-positives	0.23 [0.16, 0.29] (37/164)	0.21 [0.15, 0.28] (35/164)
False-positives	0.19 [0.13, 0.25] (31/164)	0.15 [0.10, 0.21] (25/164)
False-negatives	0.27 [0.20, 0.34] (44/164)	0.28 [0.21, 0.35] (46/164)

Data are presented as percentages with 95% confidence intervals in brackets and the number of patients or lesions shown in parentheses

*AI* artificial intelligence, *AP* average precision, *AUC* area under the receiver operating characteristic curve, *FPPI* average false-positive rate per patient

## Discussion

This study demonstrates the feasibility and safety of our AI software for csPCa detection on bpMRI in a prospective, real-world clinical setting. Feasibility was confirmed by having < 10% technical issues. Safety was corroborated by the absence of SADEs. In addition, the study explores the software’s diagnostic performance, comparing it to the PI-RADS assessment of an experienced radiologist. The software could, using the optimized threshold, reduce the number of false-positives by 5% while detecting the same number of csPCa as an expert radiologist, showing promise to minimize unnecessary biopsies while maintaining detection. However, this potential reduction should be interpreted cautiously, given the pilot design and lack of statistical power for performance outcomes.

The software functioned reliably within the clinical setting, reinforcing its potential for routine implementation [21]. Ensuring AI tools meet or exceed existing safety standards is crucial for clinical adoption [29]. No SADEs were recorded, and only three non-serious adverse events occurred in this study, none of which were caused by the software and all within the expected range for routine practice [30].

Our findings align with recent research showing that AI can match experienced radiologists in detecting csPCa cases while reducing false-positives [16]. The detection performance aligned with or surpassed previously published studies [16, 18, 31, 32], suggesting that our software may offer a competitive approach for csPCa detection. Both the software and the radiologist showed modest lesion-level sensitivities (0.43–0.46), reflecting real-world bpMRI challenges. These values likely stem from variable lesion conspicuity due to the lack of contrast, as well as biopsy confirmation difficulties, such as registration errors affecting small lesions or those in the transition zone and apex. However, these results should be interpreted with caution as the study was not specifically powered for performance evaluation. To our knowledge, only two prospective studies have evaluated similar AI software for csPCa detection, reporting patient-level sensitivities and specificities of 0.97 and 0.03, and 0.88 and 0.65, respectively [33, 34]. Both of the studies used a deep learning-based approach for lesion detection in patients already planned for biopsy. In contrast, our study enrolled all eligible patients at first contact, reducing selection bias while achieving comparable or improved detection with fewer false-positives (sensitivity and specificity of 0.92 and 0.68 with the optimized threshold).

Unlike the deep learning models commonly employed in most published studies, the AI system in our study was based on radiomics, offering a more transparent approach to PCa detection. Radiomics extracts quantitative, hand-crafted features from images, generating interpretable results that clinicians can better understand, potentially increasing trust and clinical acceptance [35, 36], which aligns with emerging regulatory frameworks like the AI Act that emphasize transparency and reliability in medical AI applications [22].

The study also highlights broader challenges in the field of AI PCa diagnostics. While most published research remains retrospective, this prospective study emphasizes the importance of real-time validation to address biases inherent in retrospective designs. Notably, the performance observed in this prospective study closely aligned with our previously published development study on retrospective data [19], reinforcing the generalizability of the software and consistency across different study designs. Prospective studies are inherently more complex, requiring adherence to pre-registered protocols, real-time decision-making, and rigorous regulatory oversight [21]. The ability to navigate these challenges while maintaining methodological integrity represents an achievement of this study.

While the AI software was evaluated as a stand-alone system against an expert radiologist, its independent use in clinical practice remains unlikely [37]. This study does not assess AI in a co-reading workflow, which may be the most effective approach. Future research should explore how AI-assisted reading could enhance diagnostic accuracy and efficiency.

This study has limitations that must be acknowledged. The single-site design and modest sample size, which is not powered for performance analysis, may limit generalizability, particularly across diverse clinical settings and patient populations. Larger, statistically powered, multi-site trials are needed to confirm these findings and validate the software’s performance. Additionally, the use of a single radiologist introduces potential bias, as inter-reader variability in prostate MRI interpretation is well documented [4]; although the protocol did not restrict the number of readers, all exams were interpreted by one experienced radiologist due to practical reasons. While pragmatic, this may limit reproducibility and generalizability, underscoring the need for multi-reader validation. Furthermore, since systematic biopsy was not performed in all MRI-negative patients, some csPCa may have been missed, potentially biasing lesion- and patient-level sensitivity estimates. Finally, although the threshold optimization was preplanned and justified, its retrospective application underscores the need for prospective validation in a sufficiently powered study to solidify the software’s clinical utility.

In conclusion, this study demonstrates that the investigated AI software is feasible, safe, and shows promise to reduce false-positive predictions in detecting csPCa on bpMRI. By achieving higher specificity and accuracy without compromising sensitivity, the software effectively reduced false-positives, underscoring its potential to minimize unnecessary biopsies and improve patient care. These results mark a step forward in integrating AI tools into clinical practice, offering a pathway to enhanced diagnostic consistency, streamlined workflows, and more efficient healthcare delivery. Future research focusing on large-scale, multi-reader, multi-site validation of co-reading systems with optimized AI and real-world implementation will be pivotal to establishing the software’s role in routine care.

## Supplementary information

ELECTRONIC SUPPLEMENTARY MATERIAL

## Data Availability

Data generated or analyzed during this study are available from the corresponding authors upon reasonable request.

## Abbreviations

AI: Artificial intelligence
AP: Average precision
AUC: Area under receiver operating characteristic curve
bpMRI: Biparametric MRI
csPCa: Clinically significant prostate cancer
FROC: Free-response receiver operating characteristic
GG: Grade group
ISUP: International Society of Urological Pathology
MDR: Medical Device Regulation
PACS: Picture archiving and communication system
PCa: Prostate cancer
PI-RADS: Prostate Imaging-Reporting and Data System
PSA: Prostate-specific antigen
ROC: Receiver operating characteristic
SADE: Serious adverse device effect
