# Supplementary material for: Prospective validation of an AI software for detecting clinically significant prostate cancer on biparametric MRI
Source: Insights Imaging. 2026 Jan 26;17:20. doi: 10.1186/s13244-025-02199-9 (PMC12834860; doi:10.1186/s13244-025-02199-9)
Supplement: Supplementary file 1 — ELECTRONIC SUPPLEMENTARY MATERIAL [file 13244_2025_2199_MOESM1_ESM.pdf]

Prospective Validation of an AI Software for Detecting Clinically Significant Prostate Cancer on Biparametric MRI
ELECTRONIC SUPPLEMENTARY MATERIAL

Study Piloting Phase

Description

The study commenced with a three-weeks piloting phase, running from 20 December 2023 to 10 January 2024, during which 13 patients were enrolled. This phase aimed to assess software performance, ensure feasibility, and refine the final study protocol before transitioning to the main study phase. Following the piloting phase, the study was temporarily paused to implement protocol refinements, resuming on 29 January 2024, with the finalized protocol. Only the results from the main study phase patients were included in the final evaluation shown in the main manuscript body. All the results from the piloting phase patients were discarded and not analyzed in the final evaluation.

The primary protocol used during the piloting phase initially set the artificial intelligence (AI) software's lesion detection threshold at 0.687. This threshold was selected based on retrospective performance analysis [1] using a dataset of 200 patients from this same study site (St. Olav's Hospital, Trondheim University Hospital, Trondheim, Norway), where it yielded approximately twice the standard-of-care false-positive rate (6% more true-negative patients being referred for targeted biopsy sampling) defined as the radiologist's false-positive rate at a PI-RADS ≥3 threshold. However, during the piloting phase, the observed false-positive rate was higher than expected. Based on these early prospective outcomes and following recommendations from the study radiologist, the threshold was adjusted to 0.73. This revised threshold more accurately achieved the intended false-positive rate target. The revised protocol, including this modification, was subsequently finalized and received approval from the ethics committee and relevant regulatory authorities before the main study phase commenced.

Additionally, a new version of the graphical user interface (GUI) was implemented at the start of the main phase. This update addressed technical failures identified during the piloting phase, which had previously hindered radiological review in select cases.

Image Acquisition

All patients underwent biparametric MRI using a 3T Magnetom Skyra MRI scanner (Siemens Healthineers, Erlangen, Germany) in accordance with PI-RADS v2.1 guidelines. Imaging was performed using a pelvic phased-array surface coil, without the use of an endorectal coil.

T2-weighted imaging (T2W) was acquired using a turbo spin-echo sequence with the following parameters: repetition time/echo time = 4760–5550/95–101 ms, field of view = 464 × 512–640 × 640 pixel^2, pixel spacing = 0.3 × 0.3 to 0.375 × 0.375 mm^2, slice thickness = 3 mm, flip angle = 160°, and number of averages = 2. Diffusion-weighted imaging (DWI) was performed using an echo-planar imaging sequence with repetition time/echo time = 4100–4400/63 ms, field of view = 128 × 128 pixels, pixel spacing = 2 × 2 mm^2, slice thickness = 3 mm, flip angle = 90°, and b-values of 50 and 800 s/mm^2. The number of averages was 2 and 8 for b = 50, and 800 s/mm^2, respectively. Apparent diffusion coefficient and DWI High b-Value maps were calculated on the scanner.

Patient Characteristics and Outcomes

Among the 13 patients, one patient withdrew consent, leaving 12 patients for analysis. The median [IQR] age was 61 [60–68] years, with a median prostate-specific antigen (PSA) level of 6.5 [3.3–9.4] ng/mL and a PSA density of 0.11 [0.07–0.25] ng/mL². Of these, 8 (67%) had a suspicious digital rectal examination, 3 (25%) had a non-suspicious examination, and 1 (8%) had unknown findings. Family history of prostate cancer was reported in 3 (25%) patients, absent in 4 (33%), and unknown in 5 (42%).

Radiological evaluation identified 9 (75%) patients as having suspicious findings: 6 (67%) were detected by both the radiologist and AI software, 3 (33%) by software alone, and none by the radiologist alone. Among these, 5 (56%) patients underwent targeted biopsies, all of whom also received systematic biopsies. The remaining 4 (44%) did not undergo biopsy as they transferred to another institution following radiological evaluation. The median [IQR] time from imaging to biopsy was 15 [14–20] days. The number of lesions detected per case was 0.5 [0–1] by the radiologist and 1 [1–2] by the software, with the highest probability score of 0.77 [0.76–0.82]. Clinically significant prostate cancer was detected in 1 patient (8%, 95% CI: [0%, 24%]).

Feasibility and Safety Outcomes

Technical issues were encountered in 3 cases (25%, 95% CI: [0%, 50%]). In one case, the radiologist found bpMRI image alignment unacceptable but was still able to delineate the AI software-detected finding. In two cases, GUI-related software deficiencies obstructed radiological review, hindering manual delineation. In one of these cases, the radiologist discarded three software-detected findings but confirmed one. In the other case, the radiologist relied on personal judgment to assess software-highlighted areas, ultimately delineating all three software findings.

No serious adverse device effects were reported.

The AI Software

The artificial intelligence (AI) system, which is described in details at [1], functions as a fully automated computer-aided detection (CAD) tool for identifying and localizing clinically significant prostate cancer (csPCa) foci on biparametric MRI (bpMRI), using a radiomics-based machine learning model. It processes T2-weighted (T2W) images, high b-value diffusion-weighted imaging (DWI), and apparent diffusion coefficient (ADC) maps. If high b-value DWI and/or ADC maps are unavailable, low b-value DWI sequences are used to synthesize the missing data using a monoexponential model.

The DWI images are co-registered to the T2W images using Elastix (v.5.0.0) [2]. Automatic segmentation of the prostate zones is performed using a 3D nnU-Net model [3] (trained using 198 cases from PROSTATEx challenge part 1 [4] and the corresponding publicly available manual segmentations by Cuocolo et al. [5]), generating whole-prostate masks. Image normalization is then applied: T2W images are N4 bias-field corrected normalized using AutoRef, a dual-tissue reference normalization method based on fat and muscle [6], while DWI and ADC images are normalized using Gaussian normalization (with ADC using fixed mean and standard deviation values).

From these images 137 features extracted, including 132 voxel-wise radiomic features are extracted using PyRadiomics (v3.0.1) [7], and 5 anatomical features. This includes:

- 94 intensity and texture features for T2W, composed of:
  - First-order statistics (n = 19),
  - Gray level co-occurrence matrix (GLCM, n = 24),
  - Gray level run length matrix (GLRLM, n = 16),
  - Gray level size zone matrix (GLSZM, n = 16),
  - Neighboring gray tone difference matrix (NGTDM, n = 5),
  - Gray level dependence matrix (GLDM, n = 14).
- 19 first-order intensity features each for high b-value DWI, and ADC images.
- 5 anatomical features from T2W images:
  - Relative distance to the prostate boundary,
  - Relative position in the x-direction,
  - Relative position in the y-direction,
  - Relative position in the z-direction,
  - Likelihood of a voxel belonging to the peripheral zone (derived from the segmentation network's softmax output).

These features are input into a pre-trained XGBoost classifier, trained on 415 patients from three datasets: PROSTATEx challenge part 1 (n = 199) [4], Prostate158 (n = 138) [8], and PCaMAP (n = 78) [9]. The model was externally validated on a separate cohort of 200 patients from this study site (St. Olav's Hospital, Trondheim University Hospital, Trondheim, Norway). These 200 patients retrospectively collected between March 2015 and December 2017 using a 3T Magnetom Skyra MRI scanner (Siemens Healthineers, Erlangen, Germany), and it has no overlapping with any of the patients scanned during this prospective study.

The classifier outputs a 3D tumor probability map, which is post-processed using morphological operations (dusting, opening, and 3D connected components detection). A fixed threshold of is applied to generate a binary lesion detection map. Only lesion candidates with local maxima exceeding the threshold are retained; all others are discarded from the final output.

For each detected lesion, SHapley Additive exPlanations (SHAP) were used to create feature importance plots, indicating the magnitude and direction of a feature's impact on the model decision for explainability.

Tables

Table S1: Image acquisition parameters used during the study (n=89).

	**T2-weighted imaging (T2W)**	**Diffusion-weighted imaging (DWI)**	**Apparent diffusion coefficient (ADC)**	**DWI High b-Value (HBV)**
Sequence type	Turbo spin-echo (89/89)	Echo planar (89/89)	Calculated on scanner (89/89)	Calculated on scanner (89/89)
Repetition time (ms)	4370 (2/89)<br>5160 (1/89)<br>5360 (82/89)<br>5560 (3/89)<br>7140 (1/89)	3700 (2/89)<br>4400 (81/89)<br>4700 (3/89)<br>5100 (1/89)<br>5400 (1/89)<br>6100 (1/89)	3700 (2/89)<br>4400 (81/89)<br>4700 (3/89)<br>5100 (1/89)<br>5400 (1/89)<br>6100 (1/89)	3700 (2/89)<br>4400 (81/89)<br>4700 (3/89)<br>5100 (1/89)<br>5400 (1/89)<br>6100 (1/89)
Echo time (ms)	97 (1/89)<br>101 (88/89)	63 (89/89)	63 (89/89)	63 (89/89)
Field of view (pixel^2)	512 × 512 (1/89)<br>640 × 640 (88/89)	120 × 128 (89/89)	120 × 128 (89/89)	120 × 128 (89/89)
Pixel spacing (mm^2)	0.300 × 0.300 (87/89)<br>0.304 × 0.304 (1/89)<br>0.390 × 0.390 (1/89)	2 × 2 (89/89)	2 × 2 (89/89)	2 × 2 (89/89)
Slice thickness (mm)	3 (89/89)	3 (89/89)	3 (89/89)	3 (89/89)
Flip angle (°)	156 (1/89)<br>159 (1/89)<br>160 (87/89)	90 (89/89)	90 (89/89)	90 (89/89)
B-values (s/mm^2)	N/A	[50, 800] (89/89)	[50, 800] (89/89)	1500 (89/89)
Number of averages	1 (1/89)<br>2 (88/89)	[2, 8] (89/89)	8 (89)	8 (89/89)

Note.—Data represent the unique values, with number of patients provided in parentheses. Joint values shown in brackets. Apparent diffusion coefficient (ADC) maps and DWI High b-Value
(HBV) images were automatically calculated and generated by the scanner. All patients scanned using a Magnetom Skyra 3T MRI scanner (Siemens Healthineers, Erlangen, Germany) with pelvic phased-array surface coil.

Table S2: Overview of the server setup and system configuration for artificial intelligence (AI) software integration in the study.

Category	Details
Server Specifications	CPU: Intel Xeon Platinum 2.6 GHz, 16 cores;<br>RAM: 32 GB;<br>Storage: 240 GB HDD;<br>GPU: NVIDIA A40-16Q
Operating System	Windows Server 2019
GUI Specifications	Version 1.0.15;<br>Based on Electron React Boilerplate
Server PACS System	Orthanc version: 1.12.1
Data Format	MRI Data DICOM format
Data Transfer Method	From Hospital: Teleradiology System via DICOM Push;<br>To GUI: HTTP requests
System Integration	DICOM integration with hospital PACS
Automation and Processing	AI software fetches scans from server PACS within one hour of receiving it from hospital PACS, processes scans fully automatically and uploads the output immediately to server PACS.
Typical Processing Time	5-10 minutes per scan

Note.—AI = artificial intelligence, CPU = central processing unit, DICOM = Digital Imaging and Communications in Medicine, GPU = graphics processing unit, GUI = graphical user interface, MRI = magnetic resonance imaging, PACS = picture archiving and communication system, RAM = random-access memory.

Table S3: Comparative analysis of patient-level results for radiologist and artificial intelligence (AI) software using the study threshold of 0.73 detection rates using histopathology as the ground truth.

Biopsy result	Radiologist	AI Software
Overall		
csPCa	69 [57, 82] (36/52)	61 [49, 73] (39/64)
Non-csPCA	31 [18, 43] (16/52)	39 [27, 51] (25/64)
Highest ISUP grade group		
<1	21 [10, 32] (11/52)	28 [17, 39] (18/64)
1	10 [2, 18] (5/52)	11 [3, 19] (7/64)
2	36 [23, 50] (19/52)	34 [23, 46] (22/64)
3	21 [10, 32] (11/52)	17 [8, 26] (11/64)
4	2 [0, 6] (1/52)	2 [0, 5] (1/64)
5	10 [2, 18] (5/52)	8 [1, 14] (5/64)

Note.—Data are presented as percentages with 95% confidence intervals in brackets and number of lesions shown in parentheses. AI = artificial intelligence, ISUP = International Society of Urological Pathology, csPCa = clinically significant prostate cancer.

Table S4: Comparative analysis of lesion-level results from the original study outcomes with software threshold of 0.73 for radiologist and artificial intelligence (AI) software detection rates using histopathology as the ground truth.

Biopsy result	Radiologist	AI Software
Overall		
csPCa	54 [43, 66] (37/68)	35 [26, 43] (40/115)
Non-csPCA	46 [34, 57] (31/68)	65 [57, 74] (75/115)
Highest ISUP grade group		
<1	29 [19, 40] (20/68)	52 [43, 61] (60/115)
1	16 [7, 25] (11/68)	13 [7, 19] (15/115)
2	29 [19, 40] (20/68)	21 [13, 28] (24/115)
3	15 [6, 23] (10/68)	9 [4, 14] (10/115)
4	3 [0, 7] (2/68)	2 [0, 4] (2/115)
5	7 [1, 14] (5/68)	3 [0, 7] (4/115)

Note.—Data are presented as percentages with 95% confidence intervals in brackets and number of lesions shown in parentheses. AI = artificial intelligence, ISUP = International Society of Urological Pathology, csPCa = clinically significant prostate cancer.

References

1 Nketiah GA, Sunoqrot MR, Sandsmark E et al (2024) Deep Radiomics Detection of Clinically Significant Prostate Cancer on Multicenter MRI: Initial Comparison to PI-RADS Assessment. Doi:10.48550/arXiv.2410.16238

2 Klein S, Staring M, Murphy K, Viergever MA, Pluim JP (2010) elastix: a toolbox for intensity-based medical image registration. IEEE Trans Med Imaging 29:196-205

3 Isensee F, Jaeger PF, Kohl SAA, Petersen J, Maier-Hein KH (2021) nnU-Net: a self-configuring method for deep learning-based biomedical image segmentation. Nat Methods 18:203-211

4 Armato SG, 3rd, Huisman H, Drukker K et al (2018) PROSTATEx Challenges for computerized classification of prostate lesions from multiparametric magnetic resonance images. J Med Imaging (Bellingham) 5:044501

5 Cuocolo R, Stanzione A, Castaldo A, De Lucia DR, Imbriaco M (2021) Quality control and whole-gland, zonal and lesion annotations for the PROSTATEx challenge public dataset. Eur J Radiol 138:109647

6 Sunoqrot MRS, Nketiah GA, Selnaes KM, Bathen TF, Elschot M (2021) Automated reference tissue normalization of T2-weighted MR images of the prostate using object recognition. MAGMA 34:309-321

7 van Griethuysen JJM, Fedorov A, Parmar C et al (2017) Computational Radiomics System to Decode the Radiographic Phenotype. Cancer Res 77:e104-e107

8 Adams LC, Makowski MR, Engel G et al (2022) Prostate158 - An expert-annotated 3T MRI dataset and algorithm for prostate cancer detection. Comput Biol Med 148:105817

9 Maas MC, Litjens GJS, Wright AJ et al (2019) A Single-Arm, Multicenter Validation Study of Prostate Cancer Localization and Aggressiveness With a Quantitative Multiparametric Magnetic Resonance Imaging Approach. Invest Radiol 54:437-447
